# Structural Correlates of Personality Dimensions in Healthy Aging and MCI

**DOI:** 10.3389/fpsyg.2018.02652

**Published:** 2019-01-08

**Authors:** Cristelle Rodriguez, Akshay Kumar Jagadish, Djalel-Eddine Meskaldji, Sven Haller, Francois Herrmann, Dimitri Van De Ville, Panteleimon Giannakopoulos

**Affiliations:** ^1^Division of Institutional Measures, Medical Direction, University Hospitals of Geneva, Geneva, Switzerland; ^2^Institute of Bioengineering, Ecole Polytechnique Fédérale de Lausanne (EPFL), Lausanne, Switzerland; ^3^Department of Electrical and Electronics Engineering, National Institute of Technology Karnataka, Surathkal, India; ^4^Institute of Mathematics, Ecole Polytechnique Fédérale de Lausanne (EPFL), Lausanne, Switzerland; ^5^Department of Radiology and Medical Informatics, University of Geneva, Geneva, Switzerland; ^6^Department of Surgical Sciences, Radiology, Uppsala University, Uppsala, Sweden; ^7^CIRD – Centre d’Imagerie Rive Droite, Geneva, Switzerland; ^8^Division of Geriatrics, Department of Internal Medicine, Rehabilitation and Geriatrics, University of Geneva, Geneva, Switzerland; ^9^Department of Psychiatry, Faculty of Medicine, University of Geneva, Geneva, Switzerland

**Keywords:** NEO personality inventory, diffusion tensor imaging, fractional anisotropy, mild cognitive impairment, canonical correlation analysis, bootstrapping

## Abstract

The revised NEO Personality Inventory (NEOPI-R), popularly known as the five-factor model, defines five personality factors: Neuroticism, Extraversion, Openness to Experience, Agreeableness, and Conscientiousness. The structural correlates of these personality factors are still a matter of debate. In this work, we examine the impact of subtle cognitive deficits on structural substrates of personality in the elderly using DTI derived white matter (WM) integrity measure, Fractional Anisotropy (FA). We employed canonical correlation analysis (CCA) to study the relationship between personality factors of the NEOPI-R and FA measures in two population groups: healthy controls and MCI. Agreeableness was the only personality factor to be associated with FA patterns in both groups. Openness was significantly related to FA data in the MCI group and the inverse was true for Conscientiousness. Furthermore, we generated saliency maps using bootstrapping strategy which revealed a larger number of positive correlations in healthy aging in contrast to the MCI status. The MCI group was found to be associated with a predominance of negative correlations indicating that higher Agreeableness and Openness scores were mostly related to lower FA values in interhemispheric and cortico-spinal tracts and a limited number of higher FA values in cortico-cortical and cortico-subcortical connection. Altogether these findings support the idea that WM microstructure may represent a valid correlate of personality dimensions and also indicate that the presence of early cognitive deficits led to substantial changes in the associations between WM integrity and personality factors.

## Introduction

Personality traits group a series of behaviors, cognitive patterns, emotional responses that characterize every single individual. They are thought to remain stable over time and may determine the social adaptation and quality of life (Huang et al., 2017). According to a widely accepted taxonomic approach, there are five major personality traits (i.e., Neuroticism, Extraversion, Openness, Agreeableness, and Conscientiousness) that are typically measured with the Neuroticism Extraversion Openness Personality Inventory-Revised (NEOPI-R), a cross-culturally validated instrument (Costa and MacCrae, 1992; McCrae et al., 1998). Neuroticism refers to the predominance of negative traits including anxiety, hostility, and anger; extraversion includes the proneness toward positive emotions and feelings such as warmth and enthusiasm; openness encapsulates the personal inclination to experience and appreciate with curious, imaginative and creative attitude new situations and thoughts; agreeableness is characterized by trustful, cooperative, and altruistic tendencies; and finally Conscientiousness qualifies the predisposition to be reliable, resolute, and well organized, and unwilling to deviate from rules and moral principles.

The structural correlates of these personality traits are still highly disputed. In spite of substantial research, most magnetic resonance imaging (MRI) investigations have led to conflicting observations due to sample heterogeneity, the variability of imaging parameters studied and age. In younger cohorts, extraversion levels have been positively associated with cortical volumes within the dorsolateral prefrontal cortex (DLPFC), inferior frontal gyrus, and temporal regions (DeYoung et al., 2010; Bjørnebekk et al., 2013) but also decreased volumes of the left occipitotemporal cortex (Li et al., 2017) as well as decreased gray matter (GM) density in middle frontal and occipitofrontal gyri (Coutinho et al., 2013). Similarly, positive associations were found between agreeableness scores and left superior temporal gyrus (Li et al., 2017), but also negative ones with left superior parietal cortex volumes (Irle et al., 2005, 2007). This NEOPI-R factor was also negatively related to GM density in the inferior parietal, middle occipital and posterior cingulate gyri (Coutinho et al., 2013). The interpretation of such correlations would be even more challenging if one considers fMRI data that has indicated that high levels of extraversion are accompanied by increased signals in DLFPC and cingulate cortex, inferior frontal gyrus, basal ganglia, thalamus, and cerebellum at rest (O’gorman et al., 2006; Kano et al., 2014). More importantly, only one Diffusion Tensor Imaging (DTI) study in younger cohorts reported worse integrity of white matter (WM) in healthy adults with high levels of neuroticism with an inverse pattern observed in respect to openness (Xu and Potenza, 2012).

The patterns of association between personality dimensions and structural parameters in contrast to younger cohorts seem to be more consistent in elderly controls. Increased cortical thickness in right superior frontal and left mesial frontal cortex were reported in cases with high levels of extraversion (Wright et al., 2007). Similar associations were found with respect to GM volumes in left temporal, dorsolateral prefrontal cortex (DLPFC) and anterior cingulate cortex. Elderly cases with high levels of agreeableness displayed an increased volume of right orbitofrontal cortex whereas the associations between cortical volumes and Conscientiousness were more variable (Kapogiannis et al., 2013). Interestingly, a lower annual rate of GM loss in right inferior parietal lobule was reported in elders with high openness scores (Taki et al., 2013). Conversely, unlike the mitigated data in younger populations, high levels of neuroticism were related to a lower thickness and GM volumes in frontotemporal cortices (Wright et al., 2007; Kapogiannis et al., 2013). Whether personality patterns have an impact on structural integrity in elderly cases with cognitive decline is still disputed. A higher anterior-posterior gradient in mesial temporal lobe atrophy was observed in Alzheimer’s Disease (AD) cases with high levels of neuroticism, but data from clinical cohorts were mostly negative (Zufferey et al., 2017). In contrast, the severity of WM lesions was strongly associated with lower levels of Conscientiousness and higher levels of neuroticism in mild cognitive impairment (MCI) cases (Duron et al., 2014).

To date, no study has addressed the relationship between personality dimensions and white matter microstructure in elderly cases prior to MCI status. Within an ongoing project focusing on the biological prediction of early cognitive changes in healthy aging, we had the opportunity to recruit a large community-based cohort of elderly individuals from the Geneva catchment area, including cognitively preserved and MCI cases, assessed with the NEOPI-R and MRI scans at baseline. The purpose of this study was to explore the association between NEOPI-R personality factors and WM microstructure in elderly controls and compare them to those observed in MCI cases. In contrast to prior studies, we studied WM changes rather than GM as various combined studies of GM and WM assessment in degenerative diseases have shown that DTI derived WM alterations are more sensitive as compared to 3D T1 derived GM alterations (Della Nave et al., 2008a,b; Bodini et al., 2009; Haller et al., 2011). We deployed DTI analysis and extracted voxelwise tract-based spatial statistics (TBSS) (Smith et al., 2006) for fractional anisotropy (FA) maps required for the analysis. In order to study its correlation with NEOPI-R personality factors, we employed Canonical Correlation Analysis (CCA) (Hotelling, 1936; Thompson, 2005; Smith et al., 2015) which is a procedure that seeks multivariate relationships between two sets of variables. The analysis was performed at the whole-brain level (Meskaldji et al., 2016) to avoid initial bias resulting from region-of-interest selection. The statistical significance of the CCA estimated the correlation between NEOPI-R personality factor and TBSS measures was determined using non-parametric permutation testing. Furthermore, we used bootstrapping strategy (Efron and Tibshirani, 1994) to find the subsets of voxels consistently contributing toward the observed correlations which were in turn highlighted in the saliency map of the brain. These maps, derived only for NEOPI-R personality factors with statistically significant correlation with TBSS measures, were then compared between our two population groups: healthy aging and MCI. Altogether our findings support the idea that the presence of early cognitive deficits led to substantial changes in the associations between WM microstructure and personality factors.

## Materials and Methods

### Participants

The research protocol was approved by the Ethics Committee of the University Hospitals of Geneva. All experimental procedures were carried out in accordance with the approved guidelines and with the principles of the Declaration of Helsinki. All participants were given written informed consent prior to inclusion. Participants were contacted via advertisements in local media to guarantee a community-based sample. Exclusion criteria included psychiatric or neurological disorders, sustained head injury, history of major medical disorders (neoplasm or cardiac illness), alcohol or drug abuse, regular use of neuroleptics, antidepressants or psycho-stimulants and contraindications to MR imaging. To control for the confounding role of cardiovascular diseases, individuals with subtle cardiovascular symptoms and a history of stroke and transient ischemic episodes were also excluded from the present study. The inclusion period for control subjects and patients with MCI was from October 2010 to March 2016.

### Neuropsychological Assessment

At baseline, all individuals underwent neuropsychological assessment. The control and MCI participants were evaluated with an extensive neuropsychological battery, including the: Mini-Mental State Examination (MMSE) (Folstein et al., 1975), the Hospital Anxiety and Depression Scale (HAD) (Zigmond and Snaith, 1983), and the Law-ton Instrumental Activities of Daily Living (IADL) (Barberger-Gateau et al., 1992). Cognitive assessment included:

(a) attention (Digit-Symbol-Coding (Wechsler, 1997), Trail Making Test A (Reitan, 1958)); (b) working memory (verbal: Digit Span Forward (Wechsler, 1997), visuospatial: Visual Memory Span (Corsi) (Wechsler, 1997)); (c) episodic memory (verbal: RI-48 Cued Recall Test (Adam et al., 2004) or RL/ RI-16 Free and Cued Recall Test (Van der Linden and Juillerat, 2004), (visual: Shapes Test (Baddeley et al., 1994)); (d) executive functions (Trail Making Test B (Reitan, 1958), and Phonemic Verbal Fluency Test (Cardebat et al., 1990)); (e) language (Boston Naming Test (Kaplan et al., 1983)); (f) visual gnosis (Ghent Overlapping Figures (Ghent, 1956)), and (g) praxis ideomotor (Schnider et al., 1997), reflexive (Poeck, 1985), and constructional (consortium to establish a registry for Alzheimers Disease, CERAD), figures copy (Welsh et al., 1994). All individuals were also evaluated with the Clinical Dementia Rating (CDR) scale (Hughes et al., 1982).

Education level was defined according to the Swiss scholar system: level 1, less than 9 years (primary school); level 2, between 9 and 12 years (high school); and level 3, more than 12 years (university). Moreover, only cases with a CDR score of 0 and scores within 1.5 standard deviations of the age-appropriate mean in all other tests were included in the control group at baseline. Participants with a CDR score of 0.5, but no dementia, and a score more than 1.5 standard deviations below the age-appropriate mean in any of the previously mentioned tests were confirmed to have MCI, in agreement with the criteria of (Petersen et al., 2001). In order to confirm the stability of the cognitive profile (and exclude cases with incipient dementia), eighteen months (2 weeks) after the baseline evaluation, control subjects underwent cognitive reassessment with the same neuropsychological battery. Participants with a performance 0.5 standard deviation lower than that at inclusion for two or more neuropsychological tests were excluded from the present study. Additionally, all individuals were clinically assessed independently by two board-certified neuropsychologists (S.T., E.T.; 4 and 2 years of experience, respectively). The final classification of controls was made blindly by a trained neuropsychologist (C.R., 10 years of experience) using both the neuropsychological scores and clinical assessment (Xekardaki et al., 2015). The demographic and clinical data have been summarized in Table 1.

**Table 1:** Demographic and neuropsychological data for the two diagnostic groups: stable controls (sCON) and Mild Cognitive Impairment (MCI).

	sCON (*N* = 163)		MCI (*N* = 57)		*P* value^∗∗^
Age, years	72.3	5.4	71.4	7.0	0.0259
**Gender (nbr)**					
Female	99.0		19.0		
Male	64.0		38.0		
**Education (nbr)**					
< 9	26.0		5.0		
9–12	66.0		25.0		
> 12	71.0		27.0		
MMSE	29.0	1.0	28.0	3.0	0.0086
IADL	8.0	0.0	8.0	0.0	0.0422
HAD Total	5.0	5.0	5.0	3.0	0.0440
Anxiety	4.0	4.0	4.0	4.0	0.0379
Depression	1.0	2.0	1.0	3.0	0.0388
Digit Span Forward	5.0	1.0	6.0	1.0	0.0483
Visual Memory Span Forward (; )	5.0	2.0	5.1	1.1	0.0414
**RI-48 Cued Recall Test**					
Immediate verbal cued recall	41.0	7.0	35.0	6.0	0.0034
Delayed cued recall	27.0	7.0	16.0	4.0	0.0009
Intrusions	2.0	2.0	3.0	4.0	0.0129
**Shapes Test**					
Total score (3 immediate recalls)	36.0	3.0	32.0	7.0	0.0190
Delayed recall	12.0	0.0	12.0	3.0	0.0138
Boston Naming Test	20.0	1.0	18.9	0.7	0.0060
Digit-Symbol-Coding	55.7	12.2	47.4	7.3	0.0069
**Trail Making Test A**					
Time,s	39.0	16.0	39.0	15.0	0.0405
Error	0.0	0.0	0.0	0.0	0.0466
**Trail Making Test B**					
Time,s	89.0	45.0	110.0	60.0	0.0147
Error	0.0	1.0	0.0	1.0	0.0500
Trail Making Test B/A (; )	2.3	1.0	2.6	1.4	0.0224
Verbal Fluency (; )	22.2	5.9	19.2	7.0	0.0172
**Praxis**					
Constructional (CERAD)	11.0	0.0	11.0	0.0	0.0319
Ideomotor transitive	10.0	1.0	9.0	0.6	0.0052
Ideomotor intransitive	20.0	0.0	19.4	0.8	0.0078
Reflexive	7.0	1.00	6.8	0.5	0.0103
Visual gnosis (Ghent)	5.0	0.0	5.0	0.1	0.0026


*Values for each group are presented as median (left-column) and inter-quartile range (right-column) unless otherwise indicated. ^∗∗^Thresholded at 0.025 according to Benjamini and Hochberg (Benjamini and Hochberg, 1995).*

### Personality Assessment

Personality features and dimensions were assessed at baseline using the French version of the NEOPI-R (Costa and MacCrae, 1992; McCrae et al., 1998). Participants were asked to complete the 240-item self-report version of the NEOPI-R questionnaire using a five-point like agreement scale. The NEOPI-R assesses 30 facets, 6 for each of the following five personality factors: Neuroticism is the tendency to feel negative emotions including anxiety, hostility, and anger; Extraversion encapsulates the proneness toward positive emotions and feelings such as warmth and enthusiasm; Openness, the personal inclination to experience and the appreciation of new situations and thoughts with a curious, imaginative and creative attitude, is defined along six facets that cover imagination (or fantasy), sense of aesthetics, emotions, and feelings, but also proactive behaviors and actions to explore and experiment beyond habits and routines, as well as intellectual curiosity, and the disposition to negotiate and discuss social, political and religious values; Agreeableness, characterized by trustful, cooperative and altruistic tendencies, and finally Consciousness, is the predisposition to be reliable, resolute and well organized, and unwilling to deviate from rules and moral principles.

### MRI Data Acquisition

MR imaging was performed on a 3T clinical routine whole-body scanner (Magnetom Trio; Siemens, Erlangen, Germany). We used a standard diffusion-weighted sequence with 30 directions isotropically distributed on a sphere, 1 B_0_ image with no diffusion weighting, 128 × 128 × 64 matrix for 1.8 × 1.8 × 2.0 mm^3^ voxel sizes. Additional sequences (3D T1 WI, T2 WI, 3D FLAIR) were acquired and analyzed to exclude brain pathologies such as ischemic stroke, subdural hematomas, or space-occupying lesions. In particular, white matter lesions were analyzed according to the Fazekas score.

### TBSS Processing

FA images were created upon brain extraction using BET (Jenkinson et al., 2005) by fitting a tensor model to the raw diffusion imaging data using FDT (Behrens et al., 2003). The FA data were then subjected to further processing using the standard procedure of TBSS (Smith et al., 2006), as described in detail in the FSL software package^1^, notably obtaining a spatial normalization of the DTI data, which is the basis for the following analyses. To summarize, each subject’s FA image was first aligned with every other subject to identify the most representative FA image. Once identified it was selected as the target FA image and was a ne-aligned into MNI152 standard space. This step was subsequently followed by transformation of every subject’s FA image to 1 × 1 × 1 mm^3^ MNI152 space using a “dual” transformation (i.e., performing a nonlinear transformation to the target FA image before doing an a ne transformation to bring it to MNI152 space). These transformed FA images, in MNI space, were averaged to obtain the mean FA image. The mean FA image was then subjected to thinning to derive the mean FA skeleton. This skeleton was thresholded at FA > 0.2 (default value) to suppress areas of low mean FA which are, generally, prone to high inter-subject variability. At last, each subject’s FA image was projected onto this derived mean FA tract skeleton by assigning the local maximum FA values in the direction perpendicular to the skeleton. The tract skeleton is the basis for voxelwise cross-subject statistics and reduces potential misregistrations as the source for false-positive or false-negative analysis results.

### Statistical Analysis

#### Personality Factors

Continuous variables at baseline were compared using one-way ANOVA and categorical variables using the Kruskal-Wallis test. Gender differences were assessed using the Chi Square test. The comparison of continuous variables between controls and MCI cases were performed using the unpaired t-test. All analyses have been conducted using STATA 14.0 (STATA Corp., College Station, Tx, United States, 2015).

#### Personality Factors and TBSS

To analyze the relationship between personality factors and TBSS measures, we first prepared two data matrices: data matrix X containing TBSS measures of size NxP, where N is the number of subjects and P the number of voxels; and data matrix Y containing the scores of the five NEOPI-R personality factors of size Nx5. The data matrix Y was further split into five different matrices each of size N x1, indexed as Y^(i)^, containing the ith personality dimension of NEOPI-R. Canonical Correlation Analysis (CCA) is a technique that looks for multivariate relationships between two sets of variables (two data matrices, X, and Y) by projecting them into a subspace formed by latent variables where their correlation is maximum. The typical use cases of CCA include data reduction (Razavi et al., 2005; Avron et al., 2013), studying relationship between datasets (Smith et al., 2015) and feature selection (Kaya and Salah, 2014; Annadani et al., 2016). In practice, the number of independent projections, called canonical variants, found by CCA is not limited to one, but K [= minfrank(X); rank(Y)g] where each subsequent projection maximizes the remaining correlation

(1) $$E_{i};\;F_{i}=argmaxCorr(XE_{i};\;YF_{i})=argmaxCorr(U_{i};\;V_{i})$$

where *i* = 1,2,… K U_i_ is the ith canonical variate of X, and V_i_ is of Y; and E_i_ and F_i_ refer to ith canonical weights that result in the canonical variates U_i_ and V_i_, respectively. In this work, we refer to E and F as the canonical weight of TBSS and personality, respectively. Similarly, U and V as canonical variates of TBSS and personality, respectively. We apply CCA separately for each personality dimension Y^(i)^ and hence, the number of canonical variates is K = 1. For convenience, we refer to E_1_, F_1_, U_1_, and V_1_ as just E, F, U, and V, respectively without subscript.

**FIGURE 1:**
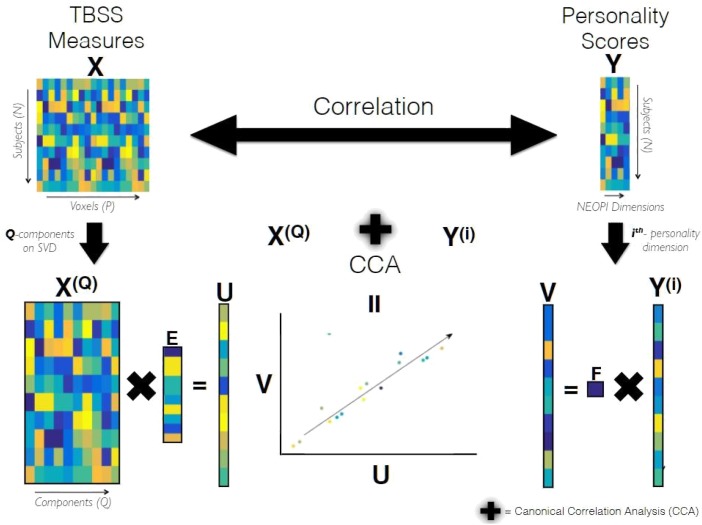
Schematic Diagram of CCA. X and Y are data matrices containing the TBSS measures and personality scores of all subjects belonging to a population group, respectively. X^(Q)^ containing the Q principle components of X, and Y^(i)^ containing scores of i-the personality dimension are subjected to Canonical Correlation Analysis (CCA). CCA yields K (= minfQ; 1g = 1) canonical variates of TBSS and personality referred as U and V, respectively. These canonical variates are maximally correlated in a subspace formed by the latent variables of X^(Q)^ and Y^(i)^. The canonical weights estimated by CCA for TBSS, referred as E, has dimensions (Q,K) and F refers to the canonical weights of personality.

CCA requires both the input matrices to be full rank but since our data matrix X has P voxels which is much larger than N (number of subjects), it was subjected to dimensionality reduction using truncated Singular Value Decomposition (SVD) to yield a dimensionality-reduced matrix X^(Q)^ of size N xQ, where Q is the number of components to be preserved determined later but it can maximally be N. The schematic diagram of CCA as used in our analysis is shown in Figure 1. The statistical significance of the correlation coefficient between matrices X^(Q)^ and Y^(i)^ estimated by CCA was tested using non-parametric permutation testing, i.e., the coefficient of the true data is compared against the distribution of coefficients obtained by randomly permuting the elements of Y^(i)^, under the assumption (null hypothesis), that no link between imaging and personality exists. The null distribution generated from 10000 different randomizations allowed us to estimate the *p*-value that indicates the statistical significance of the first canonical correlation coefficient. The optimal dimensionality Q of the data matrix X^(Q)^, mentioned earlier, was determined for each personality dimension Y^(i)^ separately by finding the component yielding the highest canonical correlation coefficient under the leave-one-out cross-validation (LOO-CV) (Lachenbruch and Mickey, 1968) scheme. The figures providing evidence for Q’s selection based on cross-validation scheme has been included in the Supplementary Material (Supplementary Figure S1).

**FIGURE 2:**
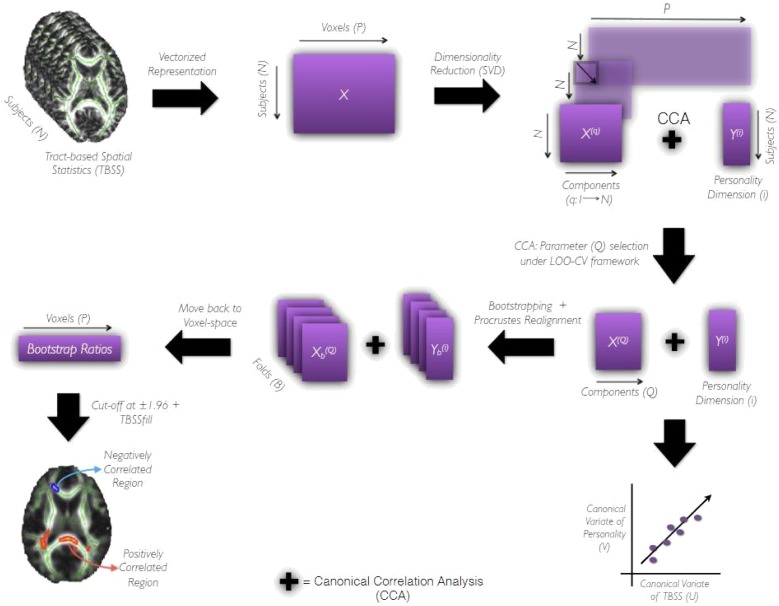
The Pipeline. It shows the processing pipeline used to derive prediction models and evaluate their performances. After data preparation as indicated by the first two blocks of the pipeline, TBSS data matrix, X^(q)^, and personality matrix, Y^(i)^ are subjected to CCA under leave-one-out cross-validation framework. The number of components preserved q ranges from (1,N). The component q yielding the highest canonical correlation coefficient under LOOCV is then chosen as the optimal component Q required for further analysis. X^(Q)^ and Y_(i)_ (both being full-data) are subjected to CCA to obtain canonical variables of personality (V) and TBSS (U) that are maximally correlated. Following which the bootstrapping strategy is employed to assess the reliability of canonical correlation coefficient (R) and canonical weights across B (= 1000) folds of the data. The canonical weights estimated for each fold are Procrustes aligned to a reference fold and used to move-back to voxel-space. Repeating the CCA followed by the back-projection step for all B folds results in a distribution of weights (having B terms) for each voxel from which the bootstrap ratio (ratio of the mean of the distribution to its standard deviation) is estimated. The sign and magnitude of this ratio indicate the type and reliability of a given voxel’s contribution toward the correlation between X^(Q)^ and Y_(i)_. The bootstrap ratios are finally thresholded at 1.96 and subjected to tbssfill operation (of FSL package) before their visualization as a saliency map.

Further, we used bootstrapping (Efron and Tibshirani, 1994) to assess the reliability of correlation between TBSS (X^(Q)^) and personality dimension (Y^(i)^), and identify the voxels consistently contributing toward it. We began by resampling subjects (with replacement) to generate B (= 1000) folds of data. Consider X_b_ and $Y_{b}^{\left(\;i\right)}$ (b = 1; 2; :::B) to indicate bth fold obtained by resampling X and Y, respectively. The data matrix X_b_ was subjected to SVD and it’s Q components were aligned to those derived from X (full-data) using Procrustes rotation (Schonemann, 1966). This step is critical because X_b_’s components tend to be rotated and reflected version of those derived from the full-data, X. More importantly, it makes the Q components of $X_{b}^{\left(\;Q\right)}$ comparable across folds (Supplementary Figures S2, S3 shows distribution of weights across both bootstrap and randomized folds). We performed CCA on $X_{b}^{\left(\;Q\right)}$ and Y^(i)^ and correct the signs of the resulting canonical weights (E_b_ and F_b_) with respect to a reference fold so that they are consistent and comparable across folds. Next, using the corrected canonical weights of TBSS (E_b_) and orthonormal matrices obtained from SVD of X_b_, we moved back from the CCA’s latent space to the voxel space. By repeating the CCA followed by back-projection step, we generated a distribution of weights having B terms for all P voxels. The bootstrap ratios were computed for each one of them by dividing the mean of their respective distribution by its standard deviation. These ratios take on real values and their sign and magnitude indicate the type and reliability of its contribution toward the correlation. We threshold the bootstrap ratio at 1:96 and pass the supra-threshold voxels through the TBSS fill function (Smith et al., 2006) of FSL for thickening the white-matter tracts. The final saliency maps were generated on FSLeyes and have the mean FA skeleton (green) and FMRIB’s 1x1x1 mm^3^ FA map (gray) as underlays. White matter tracts in the resulting saliency maps were identified and labeled using the Johns Hopkins University DTI-based white-matter atlases^2^ distributed in the FSL package. The pipeline summarizing the aforementioned steps has been shown in Figure 2. Age and gender were used as co-regressors in statistically significant models. Data are presented as unadjusted and adjusted (for age and gender effect) values.

## Results

**FIGURE 3:**
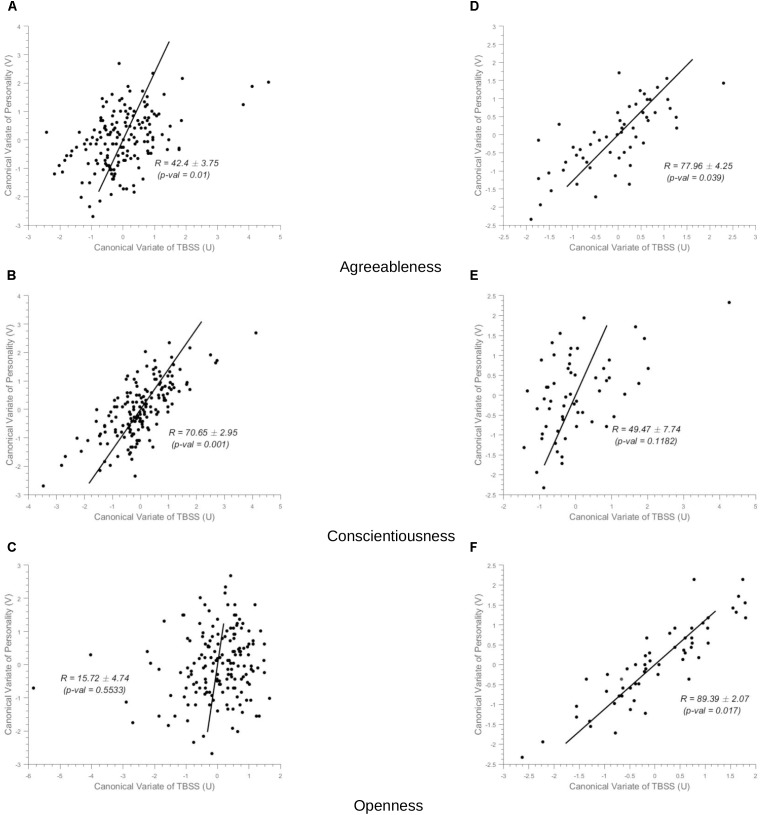
Scatter Plots. (i) Controls group (Left-Column) (ii) MCI group (Right-Column). The x-axis in the scatter plot indicates the canonical variate of TBSS (U) and the y-axis indicates canonical variate of personality (V), with dots corresponding to subjects. The canonical correlation coefficient (R) for the full-data case and the standard deviation of R computed across the 1000 bootstrap folds has been indicated in the figure with the permutation *p*-value mentioned in brackets. **(A–C)** In the left-column, are the scatter plots for Agreeableness, Conscientiousness and Openness, respectively for the sCON group (unadjusted values). **(D–F)** In the right column, correspond to Agreeableness, Conscientiousness, and Openness, respectively for the MCI group.

**FIGURE 4:**
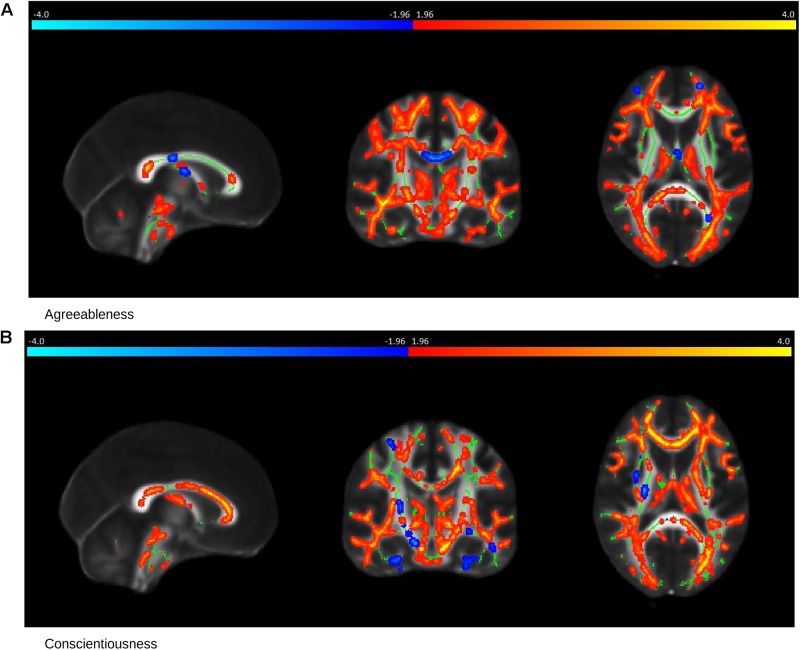
Saliency Map of the Brain for Controls group. The saliency map shows sagittal (left), coronal (middle) and axial (right) section in standard space coordinates [91, 108, 85] (radiologic convention with right hemisphere on left-hand side). Gray, mean FA value; green average skeleton. Suprathreshold voxels (whose bootstrap ratio > 1.96) after tbssfill operation (Smith et al., 2006) are highlighted in either red or yellow transition indicating positive contribution toward the correlation or in blue ! light blue indicating negative contribution toward correlation. **(A)** The prominent regions for Agreeableness in red: the sagittal striatum, splenium of corpus callosum, corona radiata, superior longitudinal fasciculus, internal capsule, and posterior thalamic radiation; in blue: body of corpus callosum, cingulate gyrus, external capsule, cortico-spinal tract, and uncinate fasciculus. **(B)** The prominent regions for Conscientiousness in red: anterior corona radiata, splenium, and genu of corpus callosum, internal capsule (anterior, posterior, and retrolenticular), sagittal striatum, and posterior thalamic radiation; in blue: external capsule, posterior, and anterior limb of internal capsule, superior longitudinal fasciculus, and posterior corona radiata of the left hemisphere.

In healthy controls, the NEOPI-R personality factors Agreeableness (*R* = 0.43, permutation *p* = 0.01, after adjustment for age and gender *R* = 0.39, permutation *p* = 0.04), and Conscientiousness (*R* = 0.71, permutation *p* = 0.001, after adjustment for age and gender *R* = 0.69, permutation *p* = 0.006) had statistically significant correlation with TBSS data. The scatter plot of these factors can be seen in Figure 3. The x and y-axis of the scatter plot corresponds to the canonical variate of TBSS (U) and personality (V), respectively. The brain saliency maps corresponding to Agreeableness and Conscientiousness of the control group have been shown in Figures 4A,B, respectively. FA values in the sagittal striatum, splenium of corpus callosum, corona radiata (anterior, posterior and superior), superior longitudinal fasciculus, internal capsule (anterior and posterior), and posterior thalamic radiation were positively related to Agreeableness. In contrast, negative correlations were found between FA values in the body of the corpus callosum, cingulate gyrus, external capsule, corticospinal tract, and uncinate fasciculus, and this personality factor. For Conscientiousness, the positive associations were present in the anterior corona radiata, splenium and genu of corpus callosum, internal capsule (anterior, posterior, and retrolenticular in the left hemisphere), sagittal striatum, and posterior thalamic radiation. Negatively associated regions included external capsule, the posterior, and anterior limb of internal capsule, superior longitudinal fasciculus and posterior corona radiata of the left hemisphere. There was no statistically significant association between the other three factors (Neuroticism, Extraversion, and Openness) of NEOPI-R and FA values in controls. For illustration purposes, we have included the scatter plot for Openness (*R* = 0.1572, permutation *p* = 0.553, after adjustment for age and gender *R* = 0.1362, permutation *p* = 0.760) in Figure 3C in order to maintain symmetry in our analysis.

**FIGURE 5:**
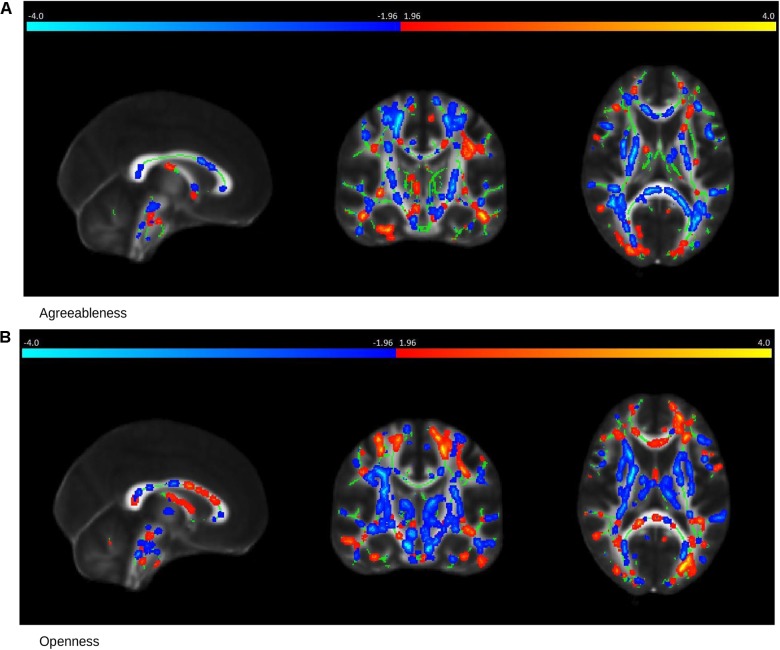
Saliency Map of the Brain for MCI group. The saliency map shows sagittal (left), coronal (middle), and axial (right) section in standard space coordinates [91, 108, 85] (radiologic convention with right hemisphere on left-hand side). Gray, mean FA value; green, average skeleton. Suprathreshold voxels (bootstrap ratio > 1.96) after tbssfill operation (Smith et al., 2006) are highlighted in either red yellow transition indicating positive contribution toward the correlation or in blue ! light blue indicating negative contribution toward correlation. **(A)** The prominent regions for Agreeableness in red: superior longitudinal fasciculus, anterior limb of internal capsule, and posterior thalamic radiation; in blue: external capsule, anterior, and superior corona radiata, posterior limb of internal capsule, sagittal striatum, splenium, and the genu of corpus callosum. **(B)** The prominent regions for Openness in red: anterior and superior corona radiata (left hemisphere), sagittal striatum (left hemisphere), retrolenticular limb of internal capsule (left hemisphere), anterior and posterior limb of internal capsule (right hemisphere), superior longitudinal fasciculus, genu of corpus callosum; in blue: the anterior and posterior limbs of the internal capsule, external capsule, posterior corona radiata, and fornix.

In MCI cases, there were strong correlations between FA values and Agreeableness (*R* = 0.78, permutation *p* = 0.04, after adjustment for age and gender *R* = 0.77, permutation *p* = 0.05) and Openness (*R* = 0.90, permutation *p* = 0.017, after adjustment for age and gender *R* = 0.90, permutation *p* = 0.014) scores whose scatter plot have been shown in Figures 3D,F, respectively. The brain saliency map corresponding to the Agreeableness dimension of the MCI group differed from that of the controls group by having a predominance of negatively related regions and is shown in Figure 5A. Positive correlations between Agreeableness scores and FA values were found in superior longitudinal fasciculus, anterior limb of internal capsule and posterior thalamic radiation (right hemisphere). Negative ones concerned the external capsule (right hemisphere), anterior and superior corona radiata (right hemisphere), posterior limb of internal capsule, sagittal striatum, splenium, and genu of corpus callosum. As seen in Figure 5B, positive associations were found between FA values and Openness scores in anterior and superior corona radiata (left hemisphere), sagittal striatum (left hemisphere), retrolenticular limb of internal capsule (left hemisphere), anterior and posterior limb of internal capsule (right hemisphere), superior longitudinal fasciculus, genu of corpus callosum. The negative associations mainly concerned the anterior and posterior limbs of the internal capsule, external capsule, posterior corona radiata, and fornix.

There was no statistically significant correlation between the other three factors (Neuroticism, Extraversion, and Conscientiousness) and FA measures in the MCI group (Figure 3E for example).

## Discussion

The present findings provide new evidence regarding the complex and partly mysterious relationships between personality and WM microstructure. In particular, they reveal that the presence of early cognitive deficits (MCI) impacts the association between WM integrity and personality factors. Agreeableness was the only factor to be associated with FA values in both MCI and controls. Openness scores were related to FA values in MCI cases, but not in controls whereas the inverse was true for Conscientiousness. Contrasting with the number of positive correlations observed in healthy brain aging, the MCI status was associated with a predominance of negative correlations indicating that higher Agreeableness and Openness scores were related to higher FA values in a limited number of cortico-cortical and cortico-subcortical connections, but also lower ones in interhemispheric and cortico-spinal tracts.

To our knowledge, this is the first study revealing a correlation between Agreeableness and WM microstructure in elderly controls. In healthy controls, Agreeableness scores were positively related to FA values in most long interhemispheric and intrahemispheric tracts including splenium of corpus callosum, corona radiata (anterior, posterior, and superior, superior longitudinal fasciculus, internal capsule (anterior and posterior), and posterior thalamic radiation). This observation is consistent with a previous study reporting a negative association between DTI mean diffusivity in corona radiata and superior longitudinal fasciculus in younger cases (Xu and Potenza, 2012). Negative associations for FA were rare and mainly concerned the body of the corpus callosum and cingulate gyrus or isolated fasciculi. In our sample, the correlations between WM microstructure and this factor were also present in MCI cases. However, the observed pattern was quite different compared to controls. Although the positive correlations persisted in this group in some tracts (in particular superior longitudinal fasciculus and internal capsule), negative associations between Agreeableness score and FA values were found in most corticospinal and intrahemispheric circuits. Altogether, these observations suggest that high Agreeableness scores are associated with better WM integrity in both interhemispheric and long intrahemispheric tracts in cognitively preserved elders. However, they also show that the presence of early cognitive deficits modifies these correlations. It is well established that MCI cases display decreased FA values in several cortical tracts in right and left frontal lobe, fornix and corpus callosum but also hippocampal connections and posterior cortical areas as a consequence of ongoing axonal damage (Stebbins and Murphy, 2009; Zhang et al., 2014). It is thus possible that MCI cases with significant WM impairment display high levels of agreeableness being more cooperative and dependent to their environment. In contrast to MCI cases with high levels of Agreeableness, those with elevated Openness scores displayed a better WM preservation in most long interhemispheric tracts including anterior and superior corona radiata, retrolenticular limb of internal capsule, anterior, and posterior limb of internal capsule but also superior longitudinal fasciculus and genu of corpus callosum. This observation should be interpreted in conjunction with the well known positive effect of high Openness in terms of cognitive trajectories in MCI cases with a lower rate of transition to clinically overt dementia (Caselli et al., 2018).

Our data add proofs supporting the association between WM status and levels of Conscientiousness. In younger cohorts, WM hyperintensities were associated with decreased scores in Conscientiousness (Booth et al., 2014). In the same line, Lewis et al. (2016) reported a positive association between better WM microstructure integrity and Conscientiousness scores in 555 older adults. However, the association between this personality factor and WM is region-dependent. For instance, high Conscientiousness levels were also associated with reductions in regional WM volume in right insula, putamen, caudate and left fusiform gyrus in women suggesting that increased expression of this personality factor may be related to regional changes in WM distribution (Liu et al., 2013). In our cohort of healthy controls, both positive and negative correlations were observed between FA and Conscientiousness values. The positive correlations concerned anterior corona radiata, splenium and genu of corpus callosum, internal capsule (anterior, posterior, and retrolenticular), sagittal striatum, and posterior thalamic radiation. In contrast, negative associations were found in external capsule, limb of internal capsule, superior longitudinal fasciculus, and left posterior corona radiata. These data indicate that high levels of Conscientiousness in old age were related to better WM integrity in some but not all long corticospinal projections (anterior corona radiata and internal capsule) as well as interhemispheric connections (splenium). In contrast, FA decrease in left corticospinal tracts is related to high scores of this personality factor as already reported by Liu et al. (2013). Importantly, DTI studies in obsessive-compulsive disorder, a clinical entity characterized by abnormally high levels of Conscientiousness also showed decreased FA values as well as increased median diffusivity in left corona radiata (Spalletta et al., 2014; Magioncalda et al., 2016; Gan et al., 2017). Altogether, these observations suggest that elderly persons with marked traits of Conscientiousness (moral rigidity, order keeping, well structured and organized thoughts with low expression of emotions) display a re-organization of WM microstructure in long intrahemispheric tracts such as corona radiata, internal and external capsule with high FA values in interhemispheric tracts.

In contrast to Conscientiousness and Agreeableness, Neuroticism, Openness, and Extraversion scores were not related to FA values in elderly controls. This contrasts with the widespread decrease of WM microstructure reported in younger cases with high levels of Neuroticism (Xu and Potenza, 2012; Bjørnebekk et al., 2013). Recent DTI studies led to conflicting data regarding the relationship between WM microstructure and Openness. This factor was positively related to FA values in tracts connecting posterior-anterior brain (Privado et al., 2017) and negatively with median diffusivity in WM adjacent to the prefrontal cortex (Xu and Potenza, 2012). Multimodal imaging analysis failed to reveal such correlations in young individuals (Bjørnebekk et al., 2013). Our data parallel this latter study showing no association between FA and Openness values in elderly controls.

From a clinical viewpoint, these results shed some new light into the structural substrates of personality dimensions in old age. They first imply that, unlike GM densities, high levels of Neuroticism have no impact on WM microstructure neither in controls nor in MCI cases. Similarly, elevated Extraversion levels, known to be associated with better preservation of GM volumes in neocortical association areas, have also no association with WM integrity in the present series. In contrast, our data reinforce the idea that, as for GM densities (Kapogiannis et al., 2013), high Agreeableness, the less studied among NEO-PI factors, is related to better WM preservation in most long interhemispheric but also intrahemispheric tracts in cognitively preserved elders. The association between Conscientiousness and WM microstructure in healthy controls is by far the more ambiguous. Mixed effects were present with better WM integrity in some among the long interhemispheric pathways such as the anterior corona radiata but also negative effects (such as those reported for the external capsule and posterior corona radiata). The possibility of a negative association between high levels of Conscientiousness and WM microstructure has been mainly documented in cases with obsessive-compulsive disorder (Spalletta et al., 2014; Magioncalda et al., 2016; Gan et al., 2017). Our findings go beyond these observations suggesting that attachment to order, moral principles and self-discipline is associated not only with region-specific increase but also decrease of WM integrity. Another main finding to be stressed is the marked differences in personality factor-FA correlations between healthy controls and MCI cases. The negative association between high Agreeableness and WM integrity as well as the preservation of several intrahemispheric tracts in MCI cases with high Openness bring the first lines of evidence supporting the attractive idea that MCI subgroups with differential vulnerability to WM damage may be defined on the basis of personality factors. Longitudinal studies focusing on the association between high levels of Agreeableness and Openness, WM damage and cognitive evolution in MCI are needed to elucidate this issue.

Strengths of the present study include the large series of community-dwelling cases with detailed neuropsychological characterization and exclusion of incipient AD cases, use of canonical correlation analysis to explore the relationships between personality and FA scores, and rigorous exclusion of cases with psychiatric disorders. Such correlation analysis differs from group comparisons since its primary objective is to investigate separately the possible relationships between our MRI parameters and personality factors in controls and MCI cases. Without *a priori* hypotheses to test, group comparisons between MCI and controls lack biological significance and should be avoided. The imaging measures, derived from TBSS, provide information on brain function and organization in each group independently of age, gender as well as other demographic and clinical parameters. Although there were large differences between CV-based (Lachenbruch and Mickey, 1968) and full-data based canonical correlation coefficients indicating the lack of homogeneity across individuals, the reliability of the correlation across bootstrap (Efron and Tibshirani, 1994) folds implies that their structure is preserved when a sufficient number of subjects are considered. Some additional limitations should be considered when interpreting the present results. First, DTI analysis was limited to FA values and does not include the remaining DTI variables such as longitudinal, radial, and mean diffusivity. Second, this is a cross-sectional study that does not explore the stability of the correlation patterns over time. Since personality factors are considered relatively stable over time, only correlations that remain stable in the long term should be retained. Last but not least, personality factors are known to be associated with a large number of MRI parameters that include gray matter measures and fMRI parameters. An integrative view that does not simply add the various significant data but forms *ad hoc* hypotheses based on them is warranted but still missing.

## Author Contributions

SH, CR, FH, and PG collected the data. DV, CR, and D-EM thought of the experiment. AJ performed the data analysis. AJ, CR, DV, and PG wrote the draft. AJ, DV, SH, CR, D-EM, and PG further edited the draft and finalized the manuscript.

## Conflict of Interest Statement

The authors declare that the research was conducted in the absence of any commercial or financial relationships that could be construed as a potential conflict of interest.

## Footnotes

1 http://www.fmrib.ox.ac.uk/fsl

2 http://www.fmrib.ox.ac.uk/fsldownloads/
