# Supplementary material for: Structural Correlates of Personality Dimensions in Healthy Aging and MCI
Source: Front Psychol. 2019 Jan 8;9:2652. doi: 10.3389/fpsyg.2018.02652 (PMC6331460; doi:10.3389/fpsyg.2018.02652)
Supplement: Supplementary file 1 [file Data_Sheet_1.pdf]

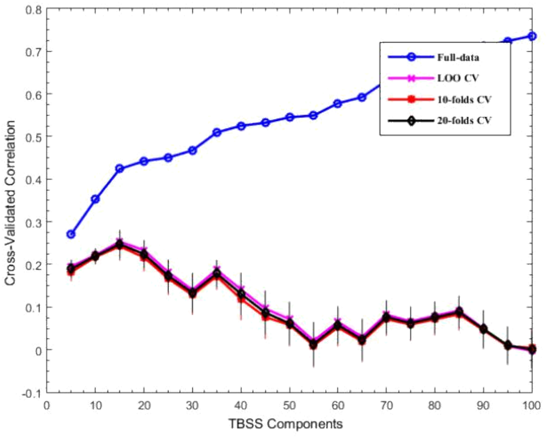

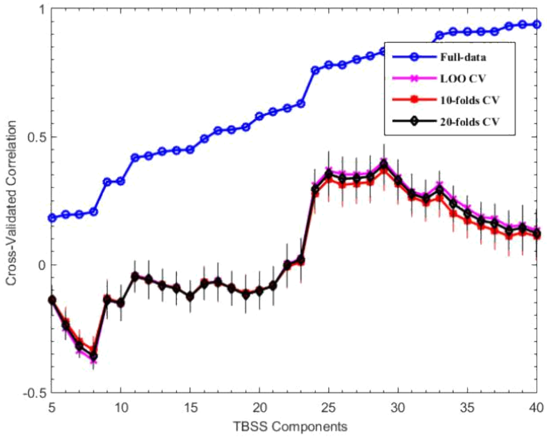

(a) Agreeableness (d)

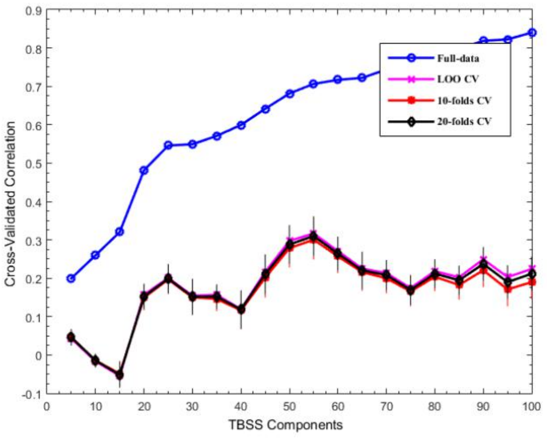

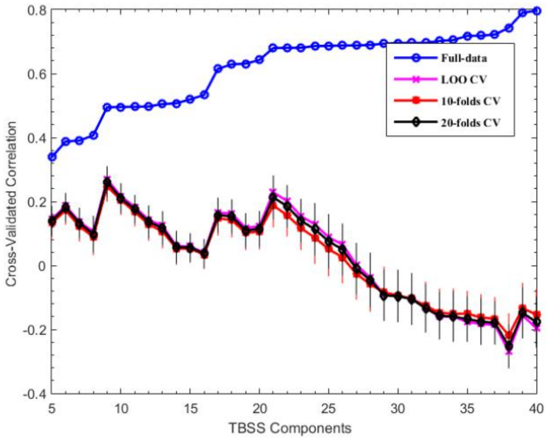

(b) Conscientiousness (e)

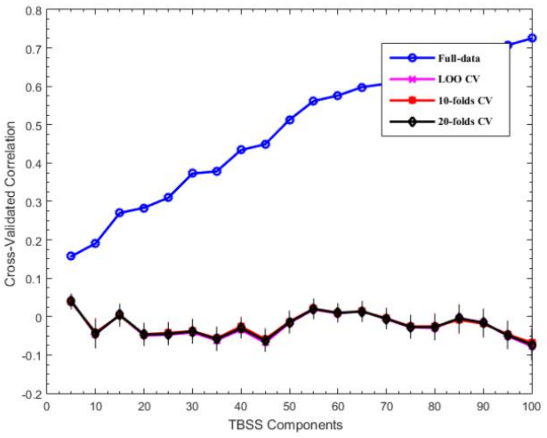

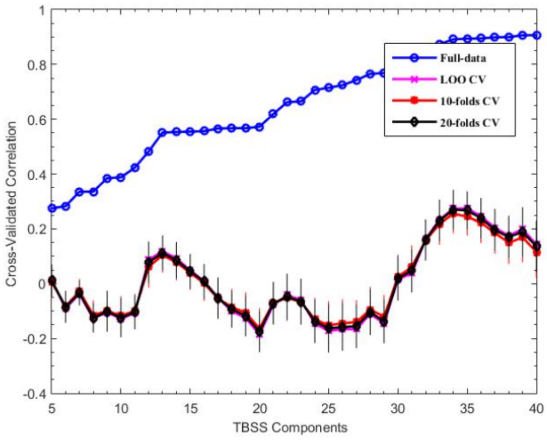

(c) Openness (f)

Figure S1: CV-based Correlation Plots. (i) Controls group (Left-Column) (ii) MCI group (Right-Column). The x-axis indicates the TBSS components (ranging 5-100 for controls; 5-40 for MCIs) and the y-axis shows the cross-validation based canonical correlation coe cient for a given personality dimension. Along with Leave-one-out cross-validation (in pink), k-fold cross validation has also been shown - k = 10 (in red) and 20 (in black). The error-bar for each component shows the 68% confidence interval generated by repeating the analysis 1000 times for a given component. These plots were used to choose the optimal number of component (Q), the one with highest correlation coe cient, for each personality factor required for further analysis. Figures (a)-(c) show the plots for Agreeableness (Q=15), Conscientiousness (Q=55) and Openness (Q=5) respectively belonging to the controls group. Whereas (d)-(f) correspond to Agreeableness (Q=25), Conscientiousness (Q=9) and Openness (Q=35) belonging to the MCI group.

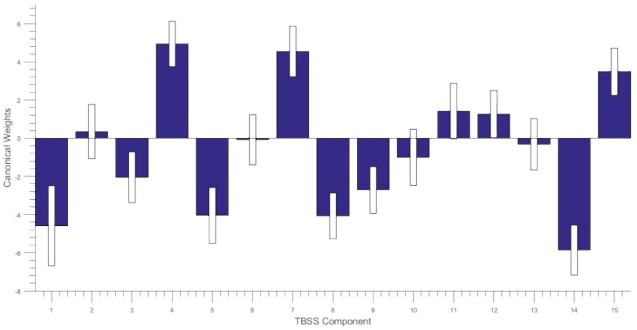

(a) Agreeableness

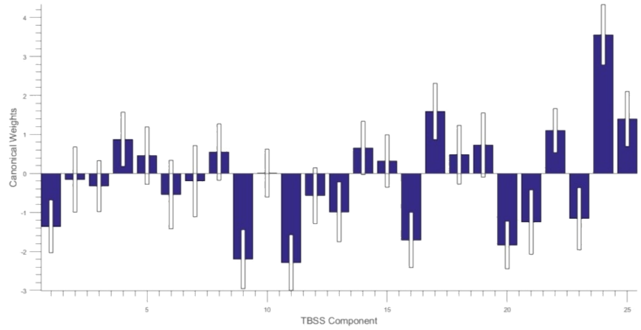

(c) Agreeableness

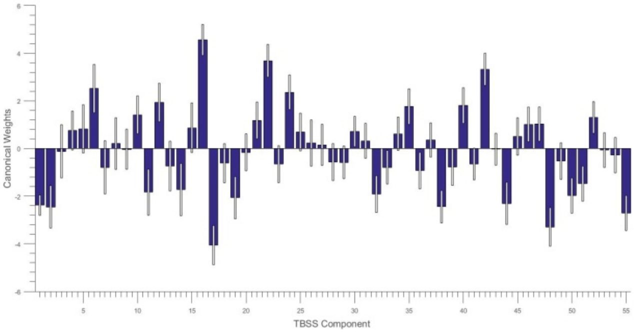

(b) Conscientiousness

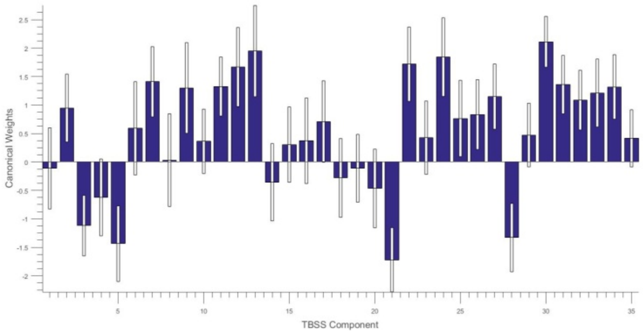

(d) Openness

Figure S2: Bootstrapping based canonical weights. (i) Controls group (Left-Column) (ii) MCI group (Right-Column). The x-axis indicates the TBSS components (q) chosen from cross-validation (Supplementary Fig. S1) and the y-axis indicates canonical weights derived by performing CCA for B (=1000) bootstrap folds of the data with the given personality dimension. The bars (in blue) indicate the mean canonical weight across the bootstrap folds for the given component and the error bars (in white) show the 68 % CI interval. Figures (a)-(b) show the variation in canonical weights for the Agreeableness and Conscientiousness measures for the controls group and Figures (c)-(d) correspond to Agreeableness and Openness measures of the MCI group. It can be seen that the canonical weights of the chosen components are fairly consistent across folds for personality factors of both the groups.

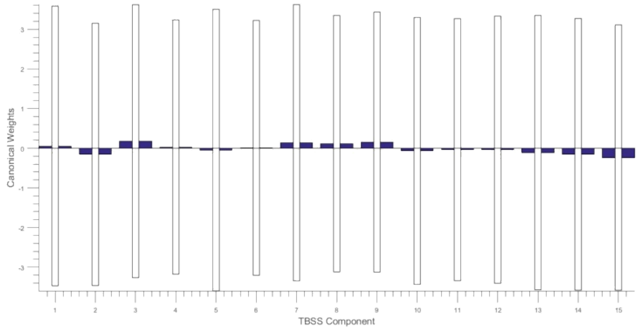

(a) Agreeableness

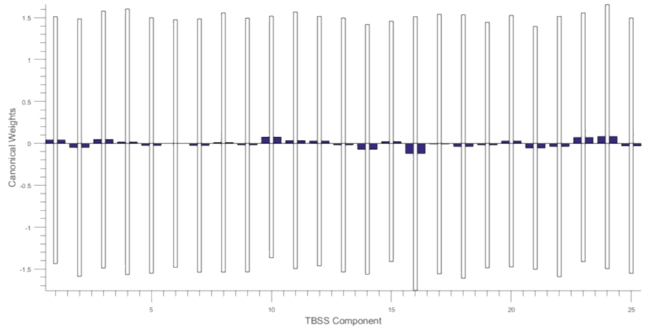

(c) Agreeableness

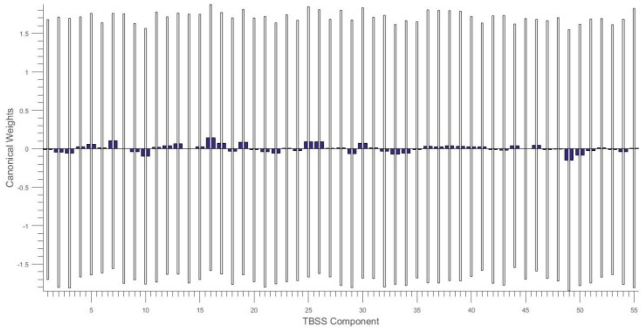

(b) Conscientiousness

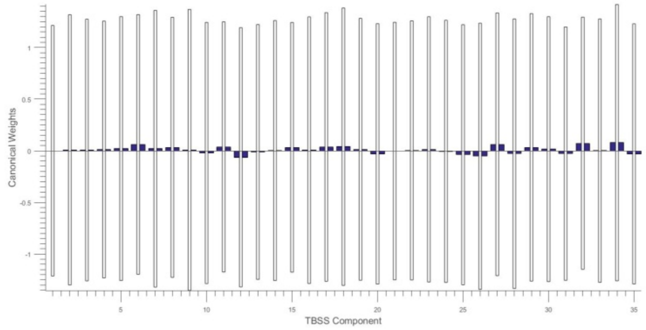

(d) Openness

Figure S3: Randomization based canonical weights.(i) Controls group (Left-Column) (ii) MCI group (Right-Column). The x-axis indicates the TBSS components (Q) chosen from cross-validation (Supplementary Fig. S1) and the y-axis indicates canonical weights obtained by performing CCA for 1000 folds of the data with the subjects of a given personality dimension permuted against the TBSS scores. It shows the distribution of canonical weights under the null hypothesis that no link exists between FA values and personality factors. The bars (in blue) indicates the mean canonical weight across the randomized folds for the given component and error bars (in white) show the 68 % CI interval. Figures (a)-(b) show the variation in canonical weights for the Agreeableness and Conscientiousness measures for the controls group and Figures (c)-(d) correspond to Agreeableness and Openness measures of the MCI group. It can be seen that the mean canonical weights for personality factors of both the groups are centered at zero with large error-bars - indicating their weak relationship under the null hypothesis.
